# Low cost production of 3D-printed devices and electrostimulation chambers for the culture of primary neurons

**DOI:** 10.1016/j.jneumeth.2015.05.001

**Published:** 2015-08-15

**Authors:** Joanna D. Wardyn, Chris Sanderson, Laura E. Swan, Massimiliano Stagi

**Affiliations:** Department of Cellular and Molecular Physiology, Institute of Translational Medicine, University of Liverpool, Crown Street, Liverpool, L69 3BX, United Kingdom

**Keywords:** 3D printer, Neuronal culture, Synapse, Axon, Dendrite, Live imaging

## Abstract

The analysis of primary neurons is a basic requirement for many areas of neurobiology. However, the range of commercial systems available for culturing primary neurons is functionally limiting, and the expense of these devices is a barrier to both exploratory and large-scale studies. This is especially relevant as primary neurons often require unusual geometries and specialised coatings for optimum growth. Fortunately, the recent revolution in 3D printing offers the possibility to generate customised devices, which can support neuronal growth and constrain neurons in defined paths, thereby enabling many aspects of neuronal physiology to be studied with relative ease. In this article, we provide a detailed description of the system hardware and software required to produce affordable 3D-printed culture devices, which are also compatible with live-cell imaging. In addition, we also describe how to use these devices to grow and stimulate neurons within geometrically constrained compartments and provide examples to illustrate the practical utility and potential that these protocols offer for many aspects of experimental neurobiology.

## Highlights

• Low cost 3D-printed devices are a cost-effective solution for live-imaging in neuronal culture.
• These devices can be used in live imaging or fixed cultures.
• Devices with constrained geometries can enrich imaging fields of view for neurites, while maintaining culture health/activity, leading to better standardisation of results.

## 1 Introduction

Although 3D printing has now been integrated into many scientific disciplines, biological researchers have only recently started to explore the potential applications of this technology. In addition to the exciting use of 3D printing to provide support structures for culturing synthetic organs (Boland et al., 2006) and tissues for regenerative medicine, there are also many possible applications of 3D printing that could benefit neuroscience. Due to the unusual geometry of neuronal cells, and the requirement for either high density cultures and/or the presence of supporting cells for optimal neuronal health; culturing primary neurons poses significant challenges for image-based experiments. Likewise, the notorious sensitivity of neurons to different surface coatings makes it difficult to easily support neuronal growth on glass, which would be the optimal choice for most imaging studies. These issues cause significant problems when trying to image physiological processes in cultured neurons. As neurons have to be supported by other cells in order to recapitulate aspects of brain physiology, a compromise is required between optimal densities for imaging and analysis of neuronal function. In particular, cell density and neuronal activity can have enormous effects on the morphology of dendritic spines (Oe et al., 2013). As a consequence, specialised and expensive devices, or microfluidic chambers, are often used to produce healthy cultures, where such features as spines and synapses can be examined in a standardised manner.

Various approaches have been taken to constrain neuronal growth by 2D patterning. 2D printing methods using modified ink-jet printers have been used to fabricate neuron-adhesive patterns such as islands and other shapes using poly(ethylene) glycol as a cell-repulsive material and collagen or poly-D-lysine (PDL) as the permissive substrate (Sanjana and Fuller, 2004). Alternatively, 2D constrained patterns of neuronal growth have also been achieved by sparsely seeding support cells, on which isolated neurons then grow to form autapses (Bekkers and Stevens, 1991). Quasi-3D growth environments have also been produced by soft lithography on polydimethyl siloxane (PDMS), to fabricate microfluidic devices for culturing neurons in etched microchannels (Kamei et al., 2015; Choi and Rogers, 2003).

If the reader is interested in a more general description of 3D printing applications and techniques and their possible use in biotechnology and organ printing we suggest the following review (Murphy and Atala, 2014). In terms of imaging cultured cells, 3D printing has mostly been used to produce scrape-tools, which are used to generate multiplexed lesions for cell migration and wound healing assays (Vitorino and Meyer, 2008). Surprisingly, the use of 3D printers to build culture dish devices for imaging neurons has not yet been explored, and there are no protocols available for making this option widely available to the scientific community.

To address this issue, we provide a detailed demonstration of the procedure to produce and utilise 3D printed devices for growing and imaging primary neuronal cultures. A range of assays were performed to establish the health and function of neurons cultured in 3D printed devices, including: K^+^ and electrical stimulation to examine synaptic vesicle recycling, axon growth assays and an analysis of the *in vitro* maintenance and creation of dendritic spines. Results from these studies show that it is possible to use 3D printing methods to produce inexpensive and adaptable devices whose physiological performance in neuronal culture is as robust as commercially available devices.

## 2 Methods

### 2.1 Assembly and set-up of 3D print stations

**Fig. 1:**
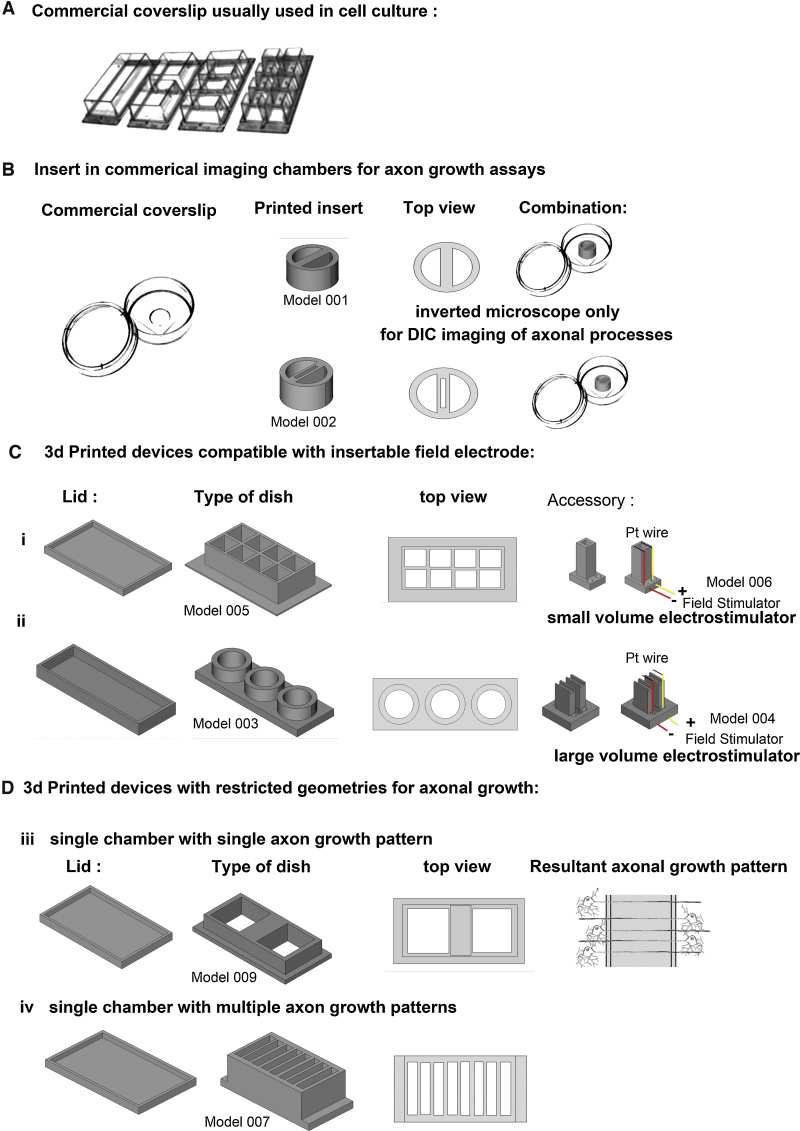
Printing different kinds of PLA dishes to culture neurons. (A) Commercial coverslips that are used in neuronal culture. (B) Combination of commercial coverslip device with 3D PLA printed adaptor (Model 001,002, Supplementary Fig. 3). This solution is ideal when we wish to convert standard culture dishes for specific needs. This particular adaptor is designed so that the central field of the culture is too low for neuronal cell bodies, thus enriching this area for neurites. The upper of the two inserts (Model 001) is for use in inverted microscopy. The lower of the two inserts (Model 002) has a viewing window in the field of neurites allowing transmission microscopy. (C) Blueprint of 3D printed coverslip: lid, 3D view, top view and mount for electrodes compatible with these dishes. (i) Eight-well chamber (Model 005) and matching electrodes (Model 006). (ii) Three-well chamber (Model 003) and accompanying electrode (Model 004). (D) (iii) Coverslip that is sealed to the glass only on the edges, the plastic wall in the middle rests on the glass surface allowing only neurites to cross over (Model 009). This system is adapted to study (in an inverted microscope) axonal transport and synaptic connections in live cultures. (iv) Device (Model 007) to produce multiple fields of neurites. Small equidistant spacers allow the culture of neurons in small volumes, allowing use of smaller volumes of media, diminishing evaporation and increasing the yield of healthy neurons.

**Fig. 2:**
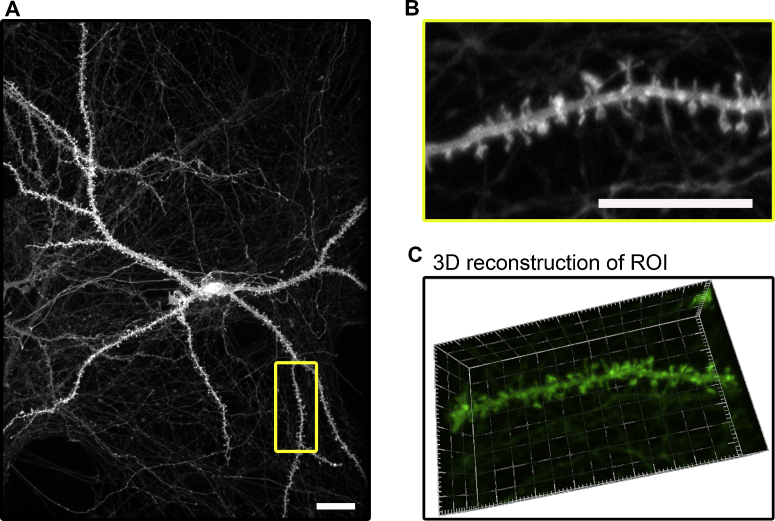
Testing 3D PLA printed device for spine morphology: (A) Hippocampal neuronal culture on PLA 3D-printed coverslip (Model 001, Model 005, Model 007). Neurons were transfected with Myr-EGFP and imaged at 21 d.i.v showing a healthy culture with spine densities and structurally indistinguishable from neurons transfected with the same vector and grown on commercial coverslips. Scale Bar: 10 μm. (B) ROI, Structure of spines at 63× in live culture. (C) 3D reconstruction of the ROI, showing the tridimensionality of spine architecture.

No particular computing facilities are required to design 3D devices. We used an Intel PC with 4GB RAM (with SD card reader). We have used a Wasp Delta 3D printer (resolution 50 μm), but any printer can use the files generated to produce your object. Currently it is possible to find several commercially available printers that are adequate to our experimental needs (when considering resolution, size and velocity). The WASP printer has the advantage that it can be operated connected to a PC but it is also a standalone system able to print from G-code (standard language code to run computerised machine tools) files from an SD card. We have used an 8GB SD Card to store G-code files ready to print. The size of this card is enough to store 1000 G-code files. Operation system: Linux Mint, Ubuntu or Debian (free of charge). We suggest these operating systems because, as the software is integrated, it is easy to install and constantly update, however all the software in this methods paper can be installed on a windows computer if preferred. To design our objects we used FreeCAD (software free of charge, http://www.freecadweb.org/): a simple CAD based on Python. The production of PLA 3D-printed objects from CAD files is extremely easy, a few steps are necessary to produce G-code files in a format which is ready to print (Fig. 1):

(1) Design your object with CAD software.
(2) Save your object in native format (if you need future modifications).
(3) Save as .vrml format if your object is finished and ready to print.
(4) Use MeshLab (software free of charge, http://meshlab.sourceforge.net/) load the .vrml format to visualise and render your 3D object. At this point you can make minor modifications if necessary. When you have explored your 3D object and the final rendering is satisfactory, save and convert to STL format.
(5) Use Cura (software free of charge, http://software.ultimaker.com/) to print directly (if your printer is attached) or generate the G-code for your 3D printer. Inside the Cura software it is possible to change the size of your objects along the three axes.
(6) Print the object using PLA plastic, preferably black to avoid possible auto-fluorescence.
(7) Remove object, and remove excess plastic with blade or sandpaper.
(8) An important step in growing neurons on glass is thorough cleaning of any glass surfaces. The glass slides to be used for making dishes are washed overnight in concentrated nitric acid, then rinsed with ample water until pH is neutral. The printed plastic forms should also be cleaned in 70% ethanol for 15 min in a bath sonicator and rinsed with deionised water. In case you have printed a coverslip device, use biocompatible glue (NEB, SYLGARD^®^ 184) to seal the dish directly to the glass cover slip (Scientific laboratory supply LTD, 22 × 50 mm) or alternatively you can use parafilm to seal the dish and commercial silicon to fix the dishes externally (for details see Supplementary Fig. 2) and sterilise the assembled device by UV irradiation. We tested both systems with success. The dish can be now coated with 0.1 mg/ml poly-D-lysine (Sigma, P0899) dissolved in 1× borate buffer (Sigma, Germany) for 1.5 h at RT or Matrigel (Becton-Dickinson Biosciences) and thoroughly washed to be ready for cell culture. During the wash you can verify the correct sealing of your dish and discard it in the rare eventuality of leaking.
(9) If you have printed a device to mount an electrode for field stimulation, thread the device with Platinum wire (Diam. 0.5 mm, 99.99% trace metals basis, Sigma, Germany) and attach the compatible plugs for connection to a Master8/DC9/GRASS S48 Stimulator or other voltage or constant current stimulator (Fig. 2C, Supplementary Fig. 3 Model 004, Model 006)

### 2.2 Spinning-disc confocal microscopy and neuronal culture

Dissociated mouse hippocampal neurons were prepared from hippocampi of C57BL/6 mice embryos (E17) as described previously (Stagi et al., 2010, 2014). Briefly: hippocampi were isolated, dispersed mechanically and seeded in culture dishes at 0.4 × 10^6^ cells/ml density. The 3D printed devices were pre-treated with poly-D-lysine (0.1 mg/ml, Sigma, Germany) or alternatively with Matrigel (Becton-Dickinson Biosciences). The cells were cultured in neurobasal medium (Invitrogen) supplemented with 2% B-27 supplement (Invitrogen) and 0.5% fetal calf serum (FCS, PAN Biotech). Cells were cultured for 20–30 days to obtain morphologically mature neurons and synapses. The plasmids vGlut1-pH (Voglmaier et al., 2006) or myristoyl-EGFP (Myr-EGFP) (Stagi et al., 2010; Um et al., 2012) were purified using the EndoFree Maxi Kit (Qiagen). Transfection was performed using electroporation at the time of dissociation using an Amaxa Nucleofector system, following the manufacturer's instructions that suggest the use of program O-005. The average transfection efficiency was from 30 to 60%, and the majority of transfected neurons showed plasmid expression from day 3 to 5 until day 30. Neurons overexpressing vGlut1-pH or Myr-EGFP were imaged at 25–30 d.i.v. at room temperature in modified Tyrode solution (pH 7.4) on a 3i Spinning Disk microscope with a Zeiss autofocus system, a motorised XY stage, Piezo Z, 6 different laser lines (405 nm/50 mW, 445 nm/40 mW, 488 nm/50 mW, 561 nm/50 mW and 640 nm/50 mW diode lasers) and Hamamatsu camera. All experiments were performed using 60× CFI Plan Apo VC, NA 1.4, oil objective and acquired at 4 frame/s (unless specified otherwise in the figure). One dendritic and one axonal arborisation were randomly selected to perform each experiment.

### 2.3 Spine analysis

Hippocampi were dissected from E17–19 mouse embryos and digested with 0.25% trypsin (37 °C; 5% CO_2_ for 10 min), and then washed with 1× HBSS and placed in plating media (Neurobasal, 2% B-27). Neurons were transfected with Myr-EGFP expression vector using an Amaxa Nucleofector, and plated on poly-D-lysine-coated (100 μg/ml) glass at 100,000 cells/well in a 3D-printed PLA dish (shown in Model 003), adhered with parafilm as described in Supplementary Fig. 2.

Between 19DIV and 23DIV, hippocampal neurons were observed with a 63× oil or 20× air objective on an inverted 3i Zeiss Spinning Disk Confocal Microscope using a 488 laser. Dendritic spine persistence or creation was assessed in consecutive images of the dendritic segments using MatLab/ImageJ software. In PLA coated dishes, spine morphology and arborisation is comparable with commercial devices as already previously described (Um et al., 2012).

## 3 Electrical stimulation

Neurons were transfected with vGlut1-pH (Voglmaier et al., 2006) and cultured on PLA covered 3D printed devices (Model 003 and Model 008, adhered to cover slips with glue). Imaging chamber inserts (3D printed), customised by the attachment of two parallel platinum wires, 5 mm apart, were used for electric field stimulation (Fig. 2C). Following the previously published methods (Voglmaier et al., 2006; Burrone et al., 2006), neurons were stimulated with 150AP at 20 Hz and images taken every 250 ms for the entire field of view.

### 3.1 Neuronal growth in custom chambers and immunocytochemistry

Neurons were cultured and plated on poly-D-lysine-coated (100 μg/ml) glass at 100,000 cells/well in a 3D printed dish (Model 009) with a shallow growth area where neuronal cell bodies are too large to fit, thus allowing the enrichment of neurites in the visualisation area (see Fig. 4). The 3D device was adhered to the glass with parafilm, to allow its later removal when processing the glass slides for immunofluorescence.

At DIV20, cortical neurons were fixed with 4% formaldehyde and 4% sucrose, permeabilised with 0.1% Triton X-100 in PBS for 30 min, and washed with PBS. The samples were then blocked with 1% BlokhenII (Aves) for 30 min, and incubated overnight at 4 °C with mouse Anti-Tubulin primary antibody at 1:500 (Promega #G7121). Primary antibodies were detected by anti-mouse Alexa-Fluor-488 conjugated secondaries and Phalloidin 555 nm was used to stain actin. Signal was stabilised with Image-It FX signal enhancer (Invitrogen). Samples were mounted with mounting solution containing DAPI (Vector Laboratories).

## 4 Results and conclusion

Plastic printing filaments are available in a variety of sizes and in different colours and materials. The most common thread diameters are 1.75 mm and 3 mm, where the smaller diameter is better adapted for finely detailed objects, and 3 mm thread is preferred for larger objects. We used 1.75 mm thread for our devices. For imaging purposes black is the natural choice to restrict autofluorescent contamination in our images. PLA (PolyLactic Acid) and Acrylonitrile butadiene styrene (ABS) are the two most commonly used desktop 3D printing materials. ABS is a high strength material well suited for medical, pharmaceutical and food packaging applications. Nevertheless, in 2010, PLA had the second highest consumption volume of any bioplastic in the world. Studies have shown that PLA is compatible with cell culture and sufficiently biocompatible to be used to build supports for vascular tissue engineering (Francesco Carfì Pavia et al., 2012; Ravi and Chaikof, 2010). It is both less expensive, and easier to work with, as less heat is required to mould this plastic for 3D printing. We chose PLA plastic for these reasons as the best alternative for building neuronal culture devices. In our setup, we printed 3D devices that were then attached to hand-coated glass coverslips for neuronal cultures. As we produce the coverslips ourselves, this would also allow the possibility to print 2D patterns on the slide to direct neuronal growth as required. To test whether these devices were suitable for long-term culture of neurons, we subjected neurons grown in various devices to some standard measures of neuronal function and health.

Dendritic spine persistence or creation was assessed in consecutive images of the dendritic segments using MatLab/ImageJ software showing that in PLA-coated dishes, the type of spine and arborisation is comparable with standard commercial devices (Um et al., 2012). When we examined neurons cultured in devices (Model 008 and Model 007) for 21 days, we found 10 ± 2 spines per 10 μm dendritic length, of which 6 ± 1 exhibited characteristic mushroom morphology, in accordance with previously published values (Fig. 2B) (Cheadle and Biederer, 2012). The quality of the culture permitted the production of 2D and 4D movies (see Supplementary Fig. 1A and B) where the spine dynamics and morphology are comparable with published data (Um et al., 2012).

**Fig. 3:**
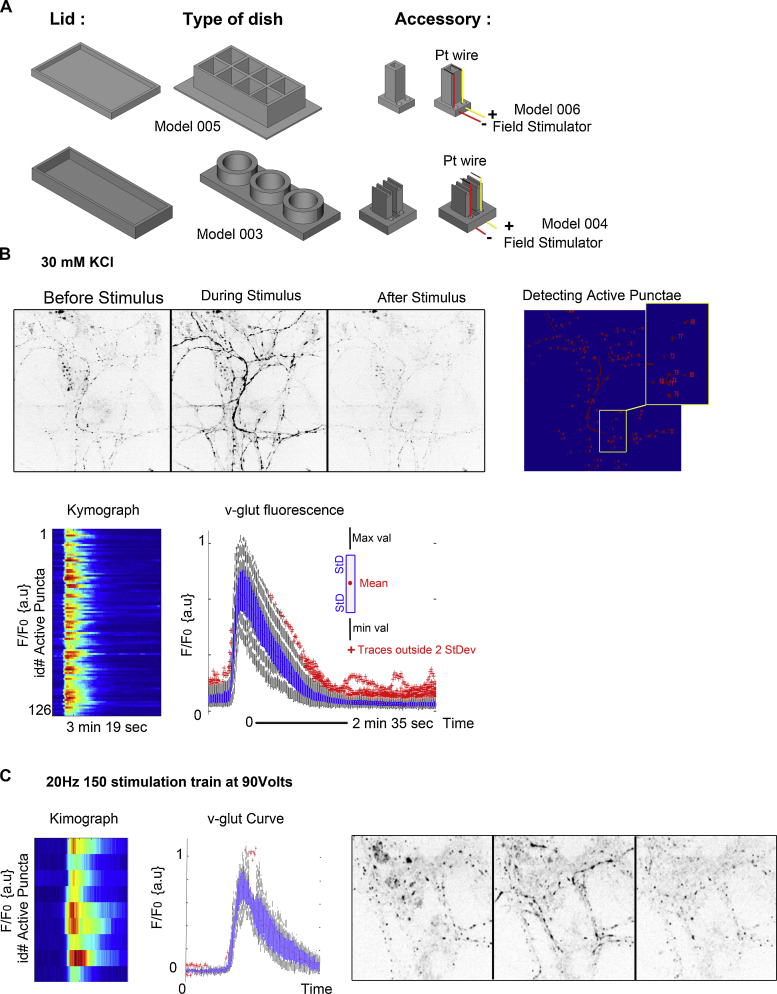
Testing 3D PLA printed devices for synaptic vesicle recycling assays: (A) 3D PLA printed device (Model 003, Model 005, Model 004,Model 006) for measuring vesicle recycling activity: (B,C) vGlut1-pHlourin assay. Neurons expressing vGlut1-pHlourin are stimulated by (B) 30 mM KCl and (C) by a train of electrical pulses using GRASS S48 Stimulator. Fluorescence increases as synaptic vesicles fuse with the plasma membrane and decreases as vesicular membrane is recovered and internalised. The colour panel shows kymographic traces of each individual synapse which responds to electrical stimulus in the field of view.

To monitor the fusion and retrieval of synaptic vesicles, we used vGlut1-pHluorin, a pH-sensitive GFP (superecliptic pHluorin) fused to vGlut1, and monitored its recycling in 21 DIV cultures, planted in device Model 003 (Model 004 electrodes to be inserted in our dishes) or Model 008 (Model 006 electrodes to be inserted in our dishes). pHluorin is quenched by the acidic pH inside a vesicle, but fluorescence emission increases when the interior of the vesicle connects with the external medium, and then rapidly declines again when the vesicle is retrieved and its interior reacidified. As synaptic vesicle recycling is sensitive to culture density and cell health, synaptic recycling assays can easily confirm that our neurons were healthy and physiologically active, and we had well defined expectations of the size and shape of the expected neuronal response. The responses, either using a 30 mM KCl stimulus (Fig. 3B) or electric field stimulation (Fig. 3C), are comparable with the natural physiology of neurons in culture, and the decay curves after stimuli are comparable again with published works (Voglmaier et al., 2006).

For our electrostimulation assays, we built electrodes to be inserted in our dishes. These devices are threaded with platinum wire and customised to the size of each imaging chamber, so that the electrode makes contact with the base of the dish. Current pulses through platinum wires generated fields of 5–10 V/cm, using a GRASS S48 Stimulator. These chambers reproduced the behaviour expected of mature synapses in culture (Voglmaier et al., 2006). A supplementary movie (Supplementary Fig. 1C) shows the full kinetics of vGlut1 recycling after electric stimulus equivalent to 150 action potentials.

**Fig. 4:**
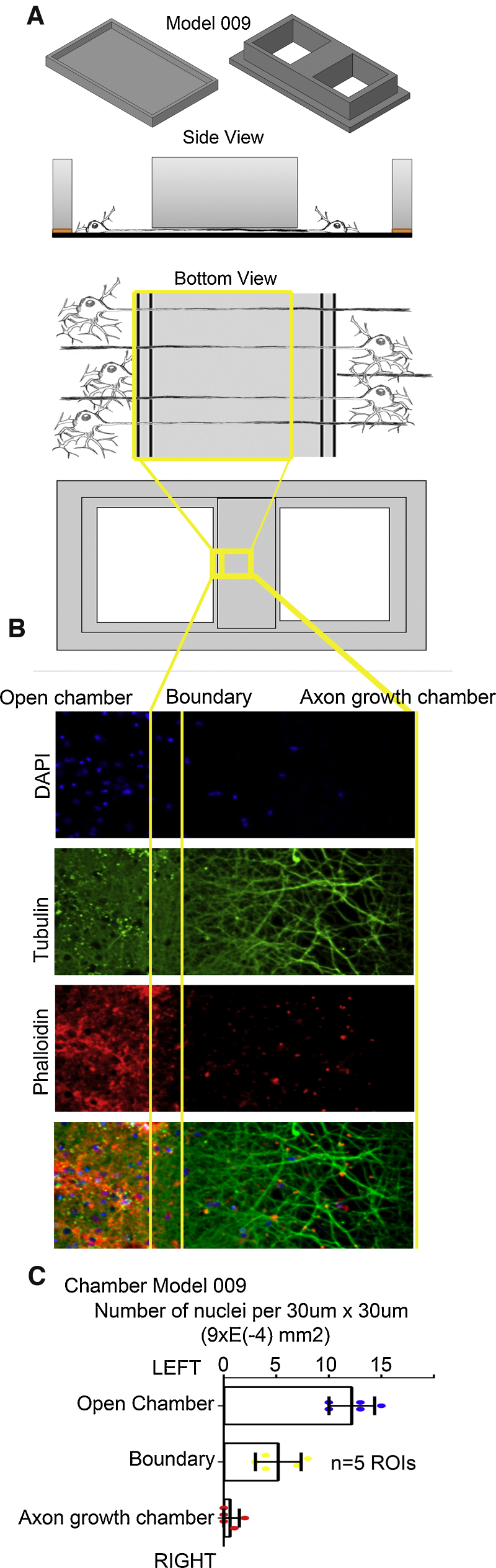
Testing 3D PLA printed devices for neurite patterning: (A) Blueprint of the coverslip. (Model 009), (B) Cultured neurons were immunostained with mouse anti-betaIII tubulin and with phalloidin for actin, and DAPI for nuclei. Images were taken with a 3i spinning disk system at 20×. The specific axon marker betaIII tubulin shows that cell bodies are excluded from a region of the coverslip by the unshielded plastic. This carpet of axons is clearly identifiable and uniform. Phalloidin staining of actin shows the distribution of growth cones on the sample. DAPI staining shows neuronal cell bodies are strongly concentrated outside the obstacle. (C) Quantification of neuronal density in Model 009 chamber. In the open field, DAPI positive nuclei were detected at about 12 per 30 μm square, whereas the central region for visualizing axons is almost completely devoid of nuclei. A border region between the open chamber and the centre of the axon visualizing region extends for approximately 200 μm, where some DAPI-positive cell bodies have migrated under the height barrier. In this boundary region, cells are about 50% the density of the open field.

We have also developed devices that allow us to enrich visual fields for axo-dendritic processes (Fig. 4). This is a particular advantage as traditional *in-vivo* methods for visualising these processes in culture require neurons to be plated at low densities to be able to clearly identify neurites. This however impairs the health and reproducibility of cultures. Our solution was to print a device with a low barrier which physically excludes neuronal cell bodies from our field of view. In this way we can exploit the health advantages of a high density culture for our studies while having clear fields of neurites to examine and/or quantify. Fig. 4 demonstrates that very few DAPI-positive cell bodies can be found in the axon visualisation area, while the area is full of beta-III tubulin-positive axonal processes and phalloidin-positive growth cones. Analysis of the density of DAPI-positive nuclei in this device showed that cell bodies are almost completely excluded from the axon visualisation region. A boundary region of about 200 μm at the edge of the axon visualisation area show the migration of some cell bodies into the restrictive zone (approximately half the density of the open field) (Fig. 4C).

We have produced and tested a series of neuronal culture devices and inserts to be used with commercially available devices. Neurons are particularly sensitive to culture conditions and typically spend weeks in culture before they can be used. For that reason, it was important to ensure that our custom-made devices are suited to long-term culture. Our plastic (PLA) is compatible with biological systems, and glue was chosen for similar reasons. Our preferred method of attachment is by using parafilm as a base and securing around the external edges—we found this to be the cheapest and least toxic method. It also has the further advantage that using the parafilm system, 3D printed devices can later be detached and samples processed for immunofluorescence.

We assayed spine formation, synaptic activity and growth of neurites and found that our systems performed at least as well as commercially available devices. Our specialised devices, where a physical constraint allows the enrichment of neurites alone in a visual field, allows us to examine neurites while maintaining a high density culture, which improves the overall health of the cells and improves the consistency of our results (Kaech and Banker, 2006).

We calculate that having purchased a 3D printer, we can produce neuronal culture devices at approximately 20% of their commercial cost, and more importantly, we have the flexibility of changing our cell culture geometry as and when required.

We have demonstrated that 3D printed dishes are compatible with primary neuronal culture, and more importantly, the cells grown in our custom-built system exhibit correct function as assessed by physiological and morphological assays. Neuronal culture can be technically demanding and expensive, however 3D printing technology opens doors to match the experimental and culture conditions to our requirements without compromising our budget. Future directions of this technology include building dishes that are permissive to 3D neuronal networks in culture, which would bring neuronal culture to a new level of modelling to understand neuronal physiology.

The use of ‘Home-made’ equipment such as these devices is not limited to neuronal culture and could be used to support growth of other cell types that may require special culture conditions, making these techniques widely applicable.
