# Supplementary figures and images for: Low cost production of 3D-printed devices and electrostimulation chambers for the culture of primary neurons
Source: J Neurosci Methods. 2015 Aug 15;251:17–23. doi: 10.1016/j.jneumeth.2015.05.001 (PMC4509708; doi:10.1016/j.jneumeth.2015.05.001)

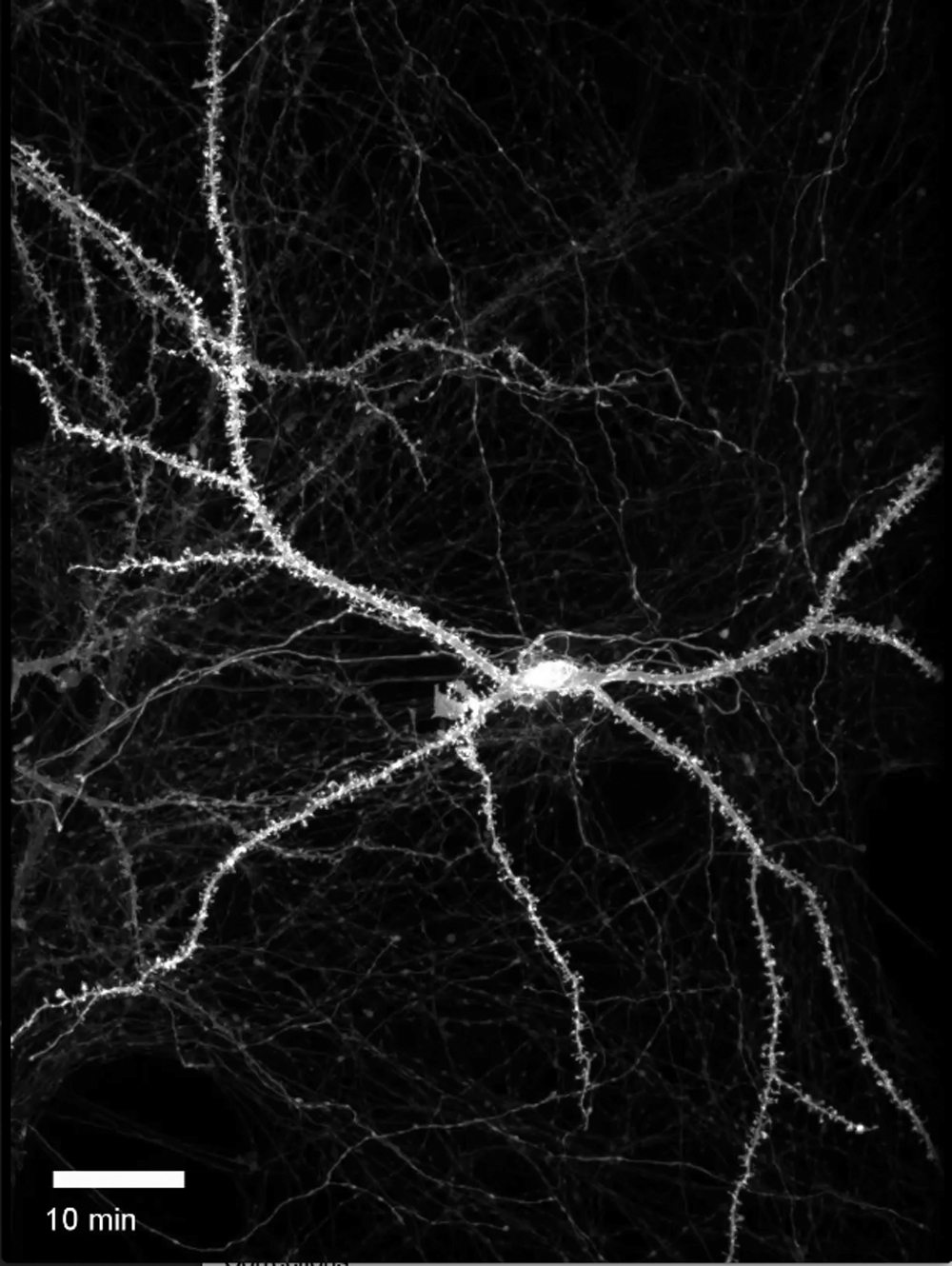

Supplement: Supplementary file 1 [file mmc1.jpg]

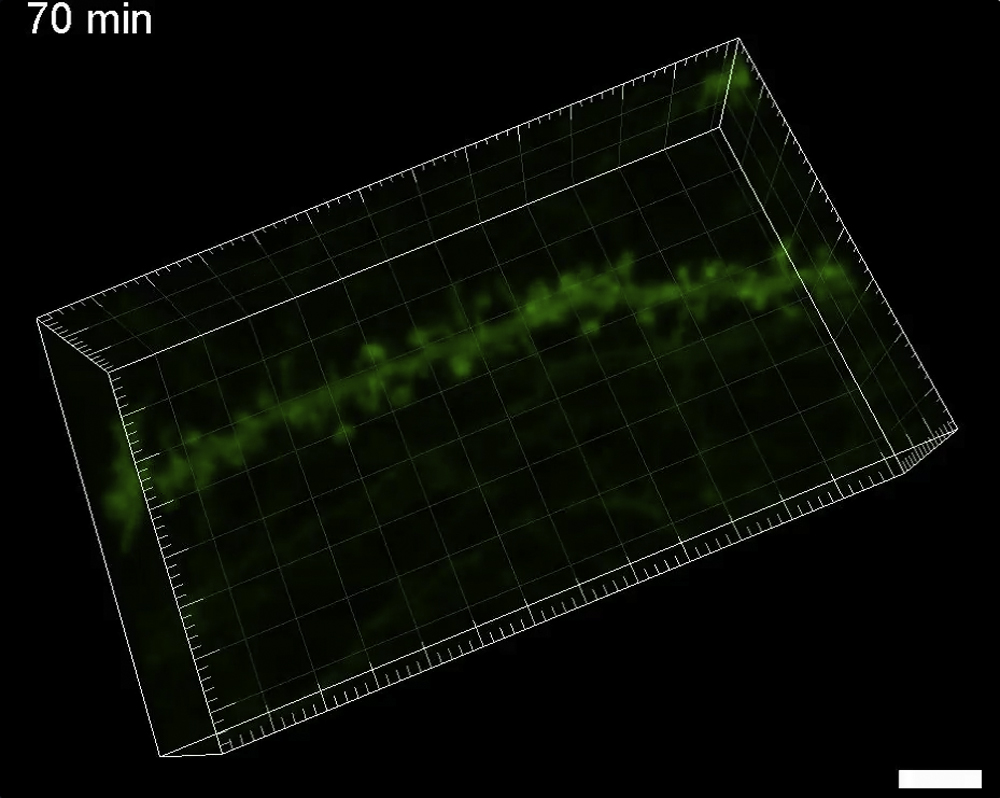

Supplement: Supplementary file 2 [file mmc2.jpg]

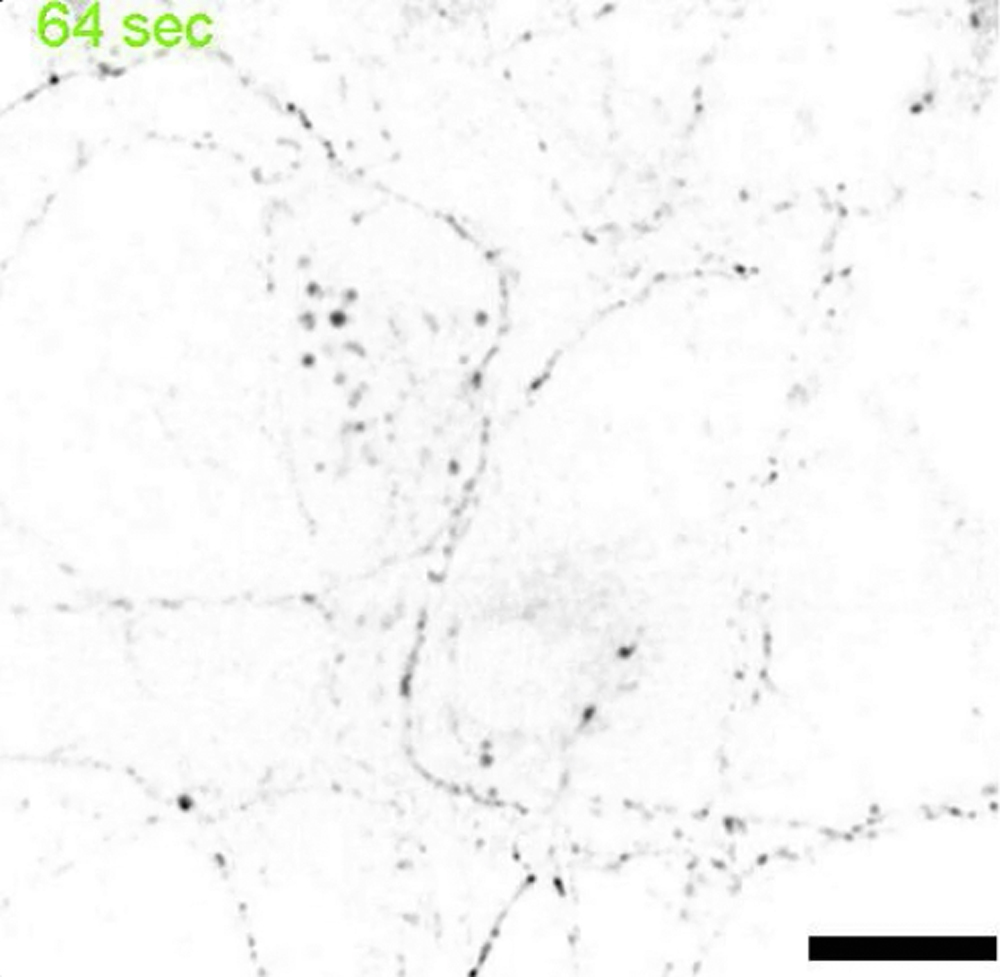

Supplement: Supplementary file 3 [file mmc3.jpg]
