# Supplementary material for: Low cost production of 3D-printed devices and electrostimulation chambers for the culture of primary neurons
Source: J Neurosci Methods. 2015 Aug 15;251:17–23. doi: 10.1016/j.jneumeth.2015.05.001 (PMC4509708; doi:10.1016/j.jneumeth.2015.05.001)
Supplement: Supplementary file 5 [file mmc5.docx]

**Supplementary materials:**

**Supplementary Figure 1.**

**
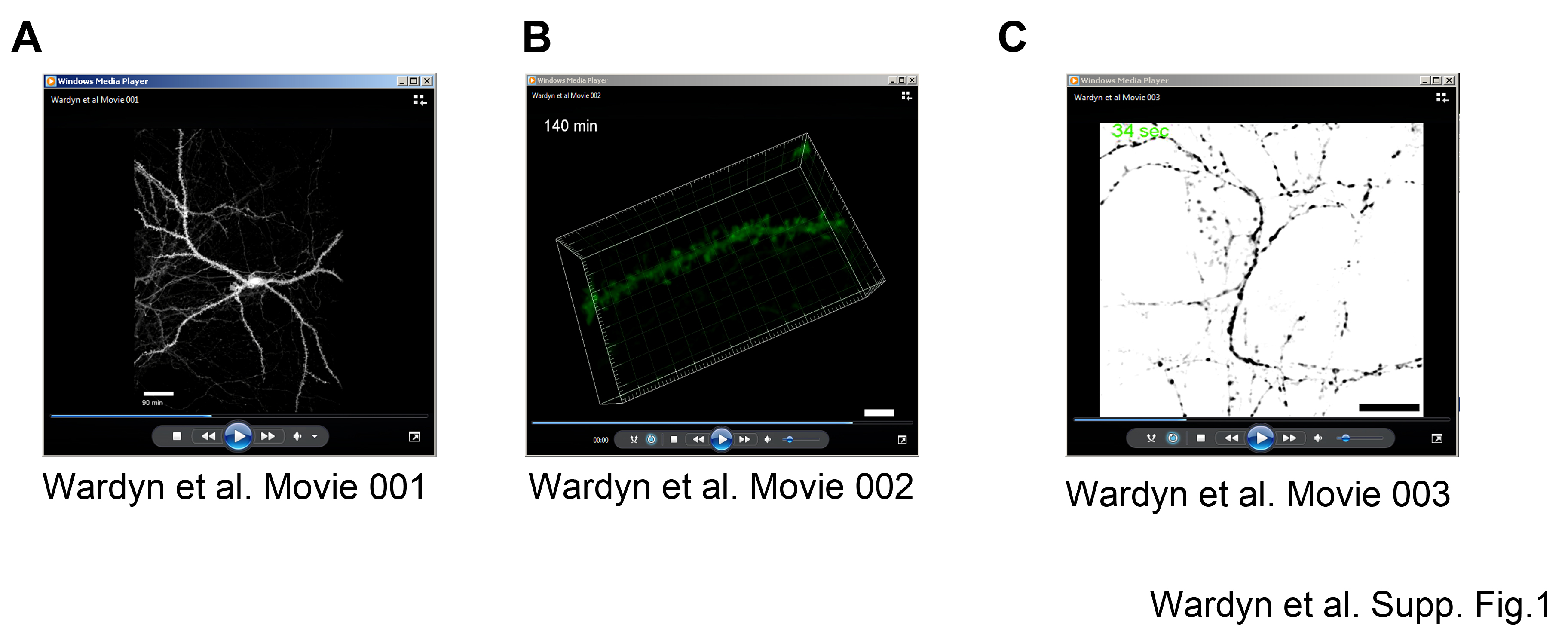
**

Avi. format Movie. For the best visualization we suggest using Windows Media Player and activating the loop function.

**Supplementary Figure 2.**

**
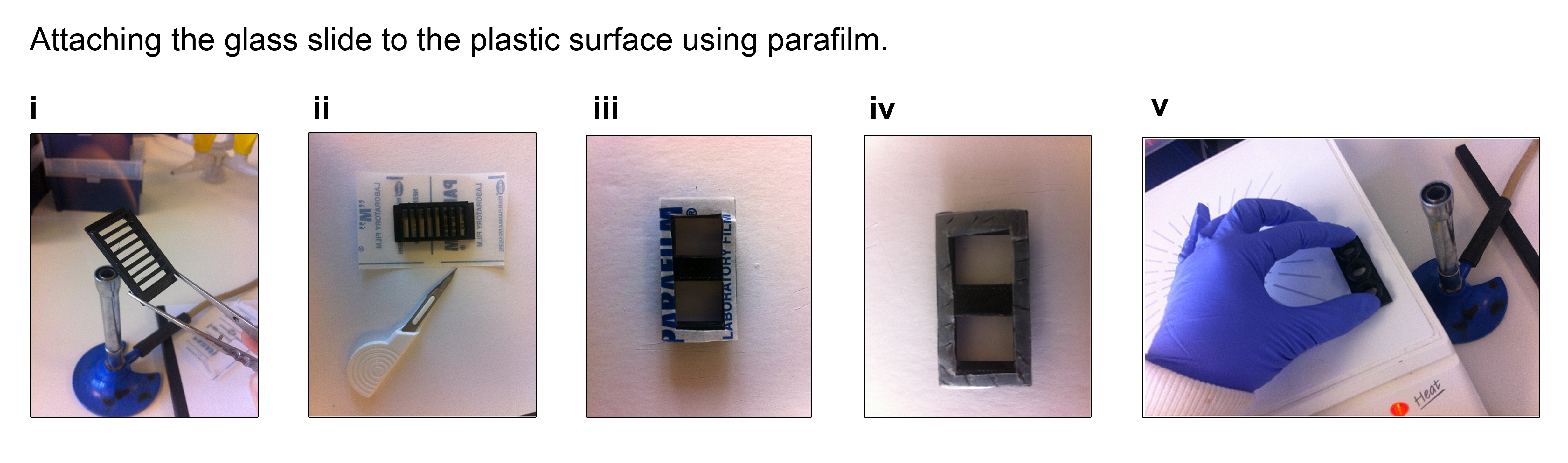
**

**Attaching the glass slide to the plastic surface using parafilm (Model 009)**

1. Occasionally there are plastic fibers visible in between the well structures as a result of printing; these can be easily burnt off using a Bunsen flame. This must be very quick to avoid bending the plastic surface of the dish
2. Cut the parafilm sheet around the dish shape with a scalpel blade.
3. Attach the parafilm to the base of the dish.
4. The parafilm layer can be attached provisionally by gently pushing it into the plastic.
5. Place the cleaned glass onto a heated stage and push the dish onto it, melting the parafilm layer in between. Be careful not to push for too long to avoid bending the plastic, which will result in media leakage.
6. The glass-side of the prepared devices are ready for UV sterilization before use.

It is important to keep the acid washed glass away from any dust so we recommend performing the assembly in the TC hood or in the presence of a Bunsen flame. As washing and gluing the dishes can be time consuming, this can be done in batches and uncoated dishes can be stored in sterile, sealed container.

**Supplementary Figure 3.**

**
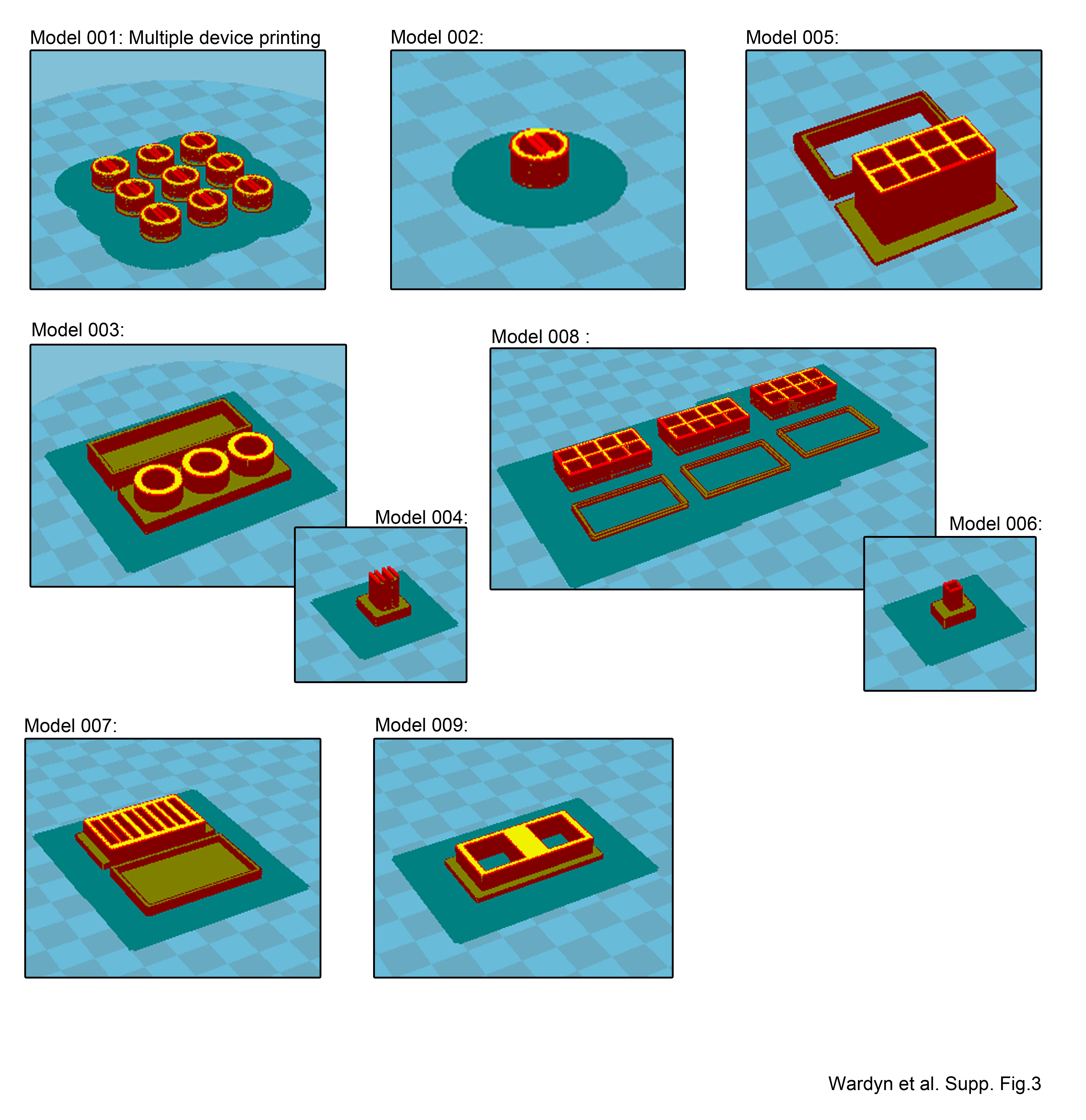
**

G-code is the common name for a numerical control (NC) programming language to control automated machine tools like 3D printers. The G-code, which can be downloaded from Journal Web Site, has been generated by Cura software (software.ultimaker.com), and is ready to print on a Wasp Delta 3D printer ([www.wasproject.it](http://www.wasproject.it)).

The following G-code is compatible with most other 3D printers, but has not been tested on printers other than the WASP Delta.

To build one’s own device, one can use free CAD software. Here we give some values that may be helpful:

Model 001: Multiple printing of Model 002.

Model 002: 8.5mm internal D, 10.5mm external D,

Model 003: 3 well of 10 mm D. External size: 57 mm x 29 mm.

Model 004: distance between the electrode: 4 mm. High: 15.0 mm

Model 005: Well cell area: 11.8 mm x 11.8 mm. Size 8 well : 57 mm x 29 mm.

External size: 72 mm x 43 mm. High: 20 mm

Model 006: distance between the electrode: 2.3 mm, external size : 4.6 mm x 4.8 mm. High: 15.3 mm

Model 007: (2.7 mm x 15.8 mm x Space 0.8 mm ) x 8 40mm x 20 mm.

Model 008: Well cell area: 11.8 mm x 11.8 mm. Size 8 well : 57 mm x 29 mm.

Model 009: 20mm X 40mm, size for axon growth 13.69 mm X 7.95 mm,

Well cell area: 13.6 mm x 13.6 mm. High: 15 mm
